# Validating virtual administration of neuropsychological testing in Parkinson disease: a pilot study

**DOI:** 10.1038/s41598-023-42934-0

**Published:** 2023-09-27

**Authors:** Julia Gallagher, Eugenia Mamikonyan, Sharon X. Xie, Baochan Tran, Sarah Shaw, Daniel Weintraub

**Affiliations:** 1 Department of Neurology, Perelman School of Medicine at the University of Pennsylvania, 3615 Chestnut St., Philadelphia, PA 19104 USA; 2 Department of Psychiatry, Perelman School of Medicine at the University of Pennsylvania, Philadelphia, PA USA; 3 Department of Biostatistics, Epidemiology and Informatics, Perelman School of Medicine at the University of Pennsylvania, Philadelphia, PA USA; 4 Parkinson’s Disease Research, Education and Clinical Center (PADRECC), Philadelphia Veteran’s Affairs Medical Center, Philadelphia, PA USA

**Keywords:** Parkinson's disease, Parkinson's disease

## Abstract

COVID-19 has highlighted the need for remote cognitive testing, but the reliability and validity of virtual cognitive testing in Parkinson disease (PD) is unknown. Therefore, we assessed PD participants enrolled in an observational, cognition-focused study with an extensive cognitive battery completed both in-person and via video conference close in time. Data for 35 PD participants with normal cognition to mild dementia were analyzed. Only one test (semantic verbal fluency) demonstrated a difference in score by administration type, with a significantly better score virtually. Only three tests demonstrated good reliability for in-person versus virtual testing, but reliability values for visit 1 versus visit 2 were similarly low overall. Trail Making Test B was successfully administered virtually to only 18 participants due to technical issues. Virtual and in-person cognitive testing generate similar scores at the group level, but with poor to moderate reliability for most tests. Mode of test administration, learning effects, and technical difficulties explained little of the low test–retest reliability, indicating possible significant short-term variability in cognitive performance in PD in general, which has implications for clinical care and research. In-person cognitive testing with a neuropsychologist remains the gold standard, and it remains to be determined if virtual cognitive testing is feasible in PD.

## Introduction

There is increasing interest in virtual (i.e., remote) administration of motor and non-motor assessments in Parkinson disease (PD), partially to allow more frequent and informative testing, as well as to minimize patient or participant burden. This trend started approximately a decade ago^1^, and was greatly accelerated by the COVID-19 pandemic, although concerns have been raised about using virtual assessments with the elderly^2^.

Cognitive assessments are a key component of clinical care and many clinical research projects, including randomized controlled trials (RCTs), yet there is little data reporting on the validity of virtual non-motor assessments in PD. If it can be determined that virtual administration of cognitive assessments is reliable and valid, the results could have a significant impact on how PD clinical care is delivered and clinical research is conducted in the future.

A systematic review indicated good reliability of virtual assessments compared with in-person assessments to diagnose dementia in general^3^, but a meta-analysis found that hetergenous data from published studies precluded interpretation, with special concern about tests that are either motor- or vision-dependent^4^. There has been one study demonstrating good retest reliability for in-person versus virtual administration of the Montreal Cognitive Assessment (MoCA) in a cohort of non-PD elderly individuals with and without cognitive impairment^5^, and a meta-analysis of cognitive testing via virtual conferencing in geriatric populations also indicated a high potential for virtual administration as a substitute for in-person testing^6^. As a note of caution, a study measuring computer literacy and its effect on both online and in-person cognitive testing suggested that older populations demonstrate worse computer literacy and perform worse on both online and in-person cognitive testing and indicated a need to correct for computer literacy when examining online cognitive test scores, specifically for tests that require motor coordination and processing speed^7^.

Although expansion of telehealth has been proposed for both clinical research^8^ and clinical care^9–12^ in PD, there has been limited assessment of virtual versus in-person traditional cognitive assessments, with studies to date either being small^13–15^ or only assessing a single global cognitive test^14,15^. As PD patients have a unique, and variable, combination of motor (e.g., tremor, slowing, rigidity), cognitive (from normal cognition to dementia), psychiatric (e.g., depression, anxiety, fatigue, daytime sleepiness and apathy), and other non-motor symptoms (e.g., a range of visual impairments), testing done in older adults or other dementia populations are not generalizable to the PD population, and a recent review of video-based visits for remote management of persons with PD highlighted the need for the validation of cognitive assessments^16^.

As an alternative to traditional, in-person, paper-and-pencil cognitive testing, remote computerized cognitive testing in various iterations is becoming increasingly common^17^. This includes unsupervised, self-completed cognitive testing, with some batteries already piloted in PD (Cogstate Brief Battery)^18^, others with previous supervised versions extensively used in PD (CANTAB Connect)^19^, and other new batteries not used yet in populations with demonstrated cognitive impairment (Amsterdam Cognition Scan)^20^. However, for the time being, supervised cognitive testing, whether traditional paper-and-pencil tests or computerized testing, remains most commonly used in clinical care and clinical research.

The objective of this study was to determine the reliability of virtual versus in-person administration of commonly used cognitive assessments in PD. We hypothesized that virtual administration of cognitive assessments would have high agreement with in-person administration, which would support virtual administration of standard cognitive assessments in the context of both clinical care and clinical research.

## Methods

### Participants

35 Parkinson’s disease patients with a range of cognitive abilities (65.7% normal cognition, 28.6% MCI and 5.7% mild dementia based on consensus diagnosis as previously outlined^21^), were recruited from the NIA U19 Clinical Core at the University of Pennsylvania (U19 AG062418). Subjects were required to have a MoCA score ≥ 20 as well as reliable internet connection to participate. Subjects were asked to complete the virtual portion of the assessment on a laptop, desktop, or tablet, although two participants completed on a smart phone due to technical issues.

### Assessments

#### Neuropsychological testing

A neuropsychologist and two research coordinators trained by the neuropsychologist administered a comprehensive neuropsychological battery assessing global cognition (using screening instruments) and the five major cognitive domains (using detailed cognitive tests). These tests are part of a research battery in a long-standing cognitive study in PD patients, many of whom have been completing these tests for years, which likely minimized practice effects between the first and second visit for this substudy. Tests administered were the MoCA (version 7.1)^22^, Mattis Dementia Rating Scale 2 (DRS-2)^23^, verbal fluency phonemic (FAS) and semantic (animals) test^24^, Hopkins Verbal Learning Test-Revised (HVLT-R)^25^, Letter-Number Sequencing (LNS)^26^, Symbol Digit Modalities Test (SDMT)^27^, Clock Drawing Test^28^, Trail Making Test A and B^29^, Judgment of Line Orientation (15-item, odd items only) (JLO)^30^ and Boston Naming Test (BNT)^31^. For the purposes of retest reliability analyses, follow-up testing was performed within 3–7 days (mean 5.37 ± 1.7 days), and order of administration type (virtual or in-person) was randomized and counterbalanced. To address possible practice effects, Form 1 and Form 4 of the HVLT-R were utilized and administered in a randomized fashion as well. A randomization schedule for administration order and HVLT-R version was created and adhered to as closely as patient scheduling allowed. In an attempt to mimic our in-person testing as closely as possible, available oral versions of tests were not administered. Finally, the same assessor completed both the virtual and in-person visit for each participant to minimize impact of inter-rater variability.

#### Virtual testing

For virtual testing, participants were asked to meet via the BlueJeans or Zoom video conferencing applications. Prior to virtual testing, participants were mailed a “virtual test packet” that included blank paper for drawing, as well as testing templates for some written tests (i.e., Trails A and B, SDMT, Clock Draw and MoCA). Test administrators shared a PowerPoint presentation that displayed relevant images and instructions that would otherwise be shown using a stimulus booklet or template to be used in conjunction with the virtual test packet (supplementary material). Some images on the PowerPoint were used for instructional purposes (e.g., SDMT, and Trails A and B), some were presented for participants to draw in their own packets (e.g., DRS-2 and MoCA), and other tests required the participant to describe what they saw on-screen (e.g., JLO, BNT and MoCA). Participants were asked to use either a laptop (N = 19), desktop (N = 6) or tablet (N = 8) to adequately view images presented to them on screen; however, two patients completed the testing via the BlueJeans or Zoom mobile app on their smartphone due to technical difficulties and reported no issues. Raters used either a desktop or laptop to administer and supervise tests.

Participants seen in-person first were provided with a stamped addressed envelope and asked to mail back the completed test packet once their virtual visit was complete. Those seen virtually first returned their virtual testing packet at their in-person visit. All virtual tests packets were returned.

#### Other assessments

Other clinical assessments included the Unified Parkinson’s Disease Rating Scale (UPDRS) Part III motor score^32^, Geriatric Depression Scale–15 Item (GDS-15)^33^, Hoehn and Yahr stage^32^ and total levodopa equivalent daily dose (LEDD)^34^. These data were collected at the first visit regardless of administration type, with exception of the UPDRS III motor score, which was obtained at the in-person visit.

#### Functional assessments

Functional assessments were administered to assess daily functioning and to assist in the consensus cognitive diagnosis process. These tests include the Penn Parkinson’s Daily Activity Questionnaire 15 (PDAQ-15)^35^ (to be completed by both patient and knowledgeable informant, if available, the Activities of Daily Living (ADLi) Questionnaire^36^ (to be completed by either knowledgeable informant (preferred) or patient), UPDRS Part II score^32^, and Schwab and England score^32^. 26 and 7 ADLIs, and 24 and 33 PDAQs, were completed by a knowledgeable informant and patient, respectively. We utilized the knowledgeable informant ADLI and patient PDAQ for analyses. These informants did not assist in completing any of the cognitive testing. These assessments were administered by the rater at the first visit, except for questionnaires completed by knowledgeable informants, which were self-completed and returned via mail.

#### Consensus cognitive diagnosis process

For descriptive purposes we provide here the consensus cognitive diagnosis (normal cognition, mild cognitive impairment or dementia) for each participant at the time the testing was done as determined by a trained panel of raters reviewing clinical and neuropsychological test data, as previously described^21^.

### Statistical analyses

Recruitment began in response to and soon after the COVID-19 pandemic onset and continued until routine in-person resumed (November 2020–August 2022). Descriptive statistics (percentages, means and standard deviations) were utilized for key demographics, cognitive tests, functional assessments, and other non-motor assessments. Paired *t* tests were used to determine the difference in average performance between in-person and virtual tests, as well as between visit one and visit two. Raw scores were used for all analyses.

Intraclass correlation coefficients (ICC) were run to assess the reliability of tests at each visit type. These were two-way mixed absolute agreement correlations, with cutoffs ≥ 0.90 being excellent, 0.75–0.9 good, 0.5–0.75 moderate, and < 0.5 poor reliability. Retest reliability based on visit number (i.e., visit 1 versus visit 2) was also examined. Finally, linear mixed-effects models (LMM) were performed to assess the effect of both administration type and visit order number on cognitive test scores. Fixed effects included administration type, visit order number, age, PD duration, education, and sex. A random intercept term was included in the mixed-effects model to account for the correlations of the cognitive scores.

Given that this is a pilot study, uncorrected *p* value < 0.05 was considered to be significant. All statistical tests are two-sided. Statistical analyses were done using SPSS (version 28^14^).

### Ethical compliance statement

This study was approved by the University of Pennsylvania IRB. All subjects provided written consent to participate in this study, which was scanned and uploaded to the Penn Integrated Neurodegenerative Disease Database. We confirm that we have read the Journal’s position on issues involved in ethical publication and affirm that this work is consistent with those guidelines.

## Results

### Participant characteristics

Descriptive information for the cohort is in Table 1. Of the 35 participants, 62.9% were male, and all were white. The mean (SD) age was 69.11 (7.79), education 16.66 (2.09) and disease duration 10.46 (5.26) years. Regarding consensus cognitive diagnosis, 23 (65.7%) had normal cognition, 10 (28.6%) mild cognitive impairment (MCI), and 2 (5.7%) dementia.

**Table 1:** Participant characteristics.

Characteristic	N	Value (Mean (SD)) or percentage	
Demographics			
Sex (% male)	35	62.9	
Age (years)	35	69.1 (7.8)	
Education (years)	35	16.7 (2.1)	
Race (% white)	35	100	
PD duration (years)	35	10.5 (5.3)	
Clinical assessments			
Total LEDD^a^ (mg/day)	35	906.5 (570.8)	
UPDRS^b^ III	35	23.3 (11.7)	
Geriatric depression scale—15 Item	35	2.6 (2.3)	
Hoehn and Yahr stage	35	2.4 (0.7)	
Cognitive assessments (in-person results)			
DRS-2^c^	35	136.1 (5.9)	
MoCA^d^	35	26.7 (2.6)	
Clock draw test	35	5.2 (1.1)	
Boston naming test	35	57.5 (3.2)	
Judgment of line orientation	35	23.3 (4.3)	
Trails A	35	42.6 (17.0)	
Trails B	35	99.5 (57.8)	
Symbol digit modalities test	35	33.9 (12.0)	
HVLT-R^e^ total immediate	35	22.2 (5.4)	
HVLT-R delayed	35	6.7 (3.7)	
HVLT-R recognition discrimination	35	9.4 (2.3)	
Letter-number sequencing	35	9.5 (2.1)	
Verbal fluency—FAS	35	45.7 (13.0)	
Verbal fluency—animals	35	18.7 (5.8)	
Cognitive diagnosis (%)	35	Normal cognition	Cognitive impairment
		65.7	34.3
Functional assessments			
Penn daily activities questionnaire—15	35	45.9 (10.7)	
Activities of daily living inventory	33	70.6 (10.2)	

^a^Levodopa equivalency daily dose.

^b^Unified Parkinson disease rating scale.

^c^Dementia rating scale-2.

^d^Montreal cognitive assessment.

^e^Hopkins verbal learning test-revised.

### Virtual versus in-person cognitive performance

#### Mean scores and t-tests

Average scores for each administration type were similar (Table 2). A paired *t* test of in-person versus virtual scores did not find statistically significant differences for mode of administration for any of the cognitive tests except the semantic verbal fluency test (*p* = 0.01) (Table 2). Virtual testing on average took slightly longer (mean time = 66.1 ± 10.11 min) to complete than in-person testing (mean time = 56.0 ± 8.4 min).

**Table 2:** Paired *t* test of in-person and virtual cognitive test scores.

Test	N	Average change (SD)^a^	t	*df*	Two-sided *p* value
DRS-2	34	− 0.88 (3.013)	− 1.708	33	0.10
MoCA	35	− 0.23 (2.250)	− 0.601	34	0.55
Clock draw test	35	− 0.37 (1.352)	− 1.625	34	0.11
Boston naming test	35	− 0.69 (2.207)	− 1.838	34	0.08
Judgment of line orientation	35	− 0.97 (5.623)	− 1.022	34	0.31
Trails A	32	− 3.69 (15.249)	− 1.368	31	0.18
Trails B	19	2.58 (32.016)	0.351	18	0.73
Symbol digit modalities test	35	− 1.46 (13.086)	− 0.660	34	0.51
HVLT-R total immediate	35	0.71 (5.050)	0.837	34	0.41
HVLT-R delayed	35	− 0.43 (3.822)	− 0.663	34	0.51
HVLT-R recognition discrimination	35	0.23 (2.263)	0.597	34	0.55
Letter-number sequencing	35	0.31 (2.285)	0.814	34	0.42
Verbal fluency—FAS	35	− 2.26 (8.511)	− 1.569	34	0.13
Verbal fluency—Animals	35	− 1.77 (3.904)	− 2.684	34	0.01

^a^Change is in-person minus virtual test performance.

Not all assessments could be completed successfully at virtual visits. Only 32 (91.4%) and 19 (54.3%) participants were able to successfully complete written Trails A and B, respectively. Administrators were unable to correct participants as per test instructions at the time of test administration, those who could not complete either of the written Trail making tests were marked as having an “administration error”.

#### Reliability

Intraclass correlations for virtual versus in-person testing demonstrated good reliability only for the DRS-2 (0.849), Trails A (0.754), and phonemic verbal fluency (0.815) (Table 3). The remaining 11 test scores showed poor or moderate reliability.

**Table 3:** Intraclass correlations of in-person and virtual test scores.

Test	N	In-person mean (SD)	Virtual mean (SD)	Correlation coefficient	Interpretation of reliability	95% CI
DRS-2	34	136.2 (5.9)	137.1 (5.3)	0.849	Good	0.718–0.922
MoCA	35	26.7 (2.6)	27.0 (2.5)	0.606	Moderate	0.345–0.779
Clock draw test	35	5.2 (1.1)	5.5 (1.0)	0.150	Poor	− 0.170 to 0.449
Boston naming test	35	57.5 (3.1)	58.1 (2.5)	0.683	Moderate	0.457–0.826
Judgment of line orientation	35	23.3 (4.31)	24.2 (5.26)	0.317	Poor	− 0.011 to 0.584
Trails A	32	42.6 (17.4)	46.3 (25.7)	0.754	Good	0.557–0/871
Trails B	19	79.3 (45.7)	76.7 (32.5)	0.685	Moderate	0.341–0.866
Symbol digit modalities test	35	33.9 (12.0)	35.3 (15.5)	0.559	Moderate	0.281–0.750
HVLT-R total immediate	35	22.2 (5.4)	21.5 (4.9)	0.516	Moderate	0.226–0.722
HVLT-R delayed	35	6.7 (3.7)	7.1 (3.4)	0.416	Poor	0.099–0.656
HVLT-R recognition discrimination	35	9.4 (2.3)	9.2 (1.8)	0.403	Poor	0.082–0.647
Letter-number sequencing	35	9.5 (2.1)	9.2 (2.1)	0.414	Poor	0.098–0.654
Verbal fluency—FAS	35	45.7 (13.0)	47.9 (15.4)	0.815	Good	0.665–0.902
Verbal fluency—animals	35	18.7 (5.8)	20.4 (5.8)	0.745	Moderate	0.518–0.686

#### Linear mixed-effects models

To further explore the impact of administration type on cognitive test performance, and to control for important covariates, we ran linear mixed-effects models. Fixed effects were administration type (in-person versus virtual), visit order number (first versus second visit), sex, age at test, PD duration and years of education. Only the semantic verbal fluency test (*p* = 0.01) proved to be significantly impacted by mode of administration, with significantly better scores for virtual versus in-person administration (Table 4).

**Table 4:** Linear mixed-effects models of in-person versus virtual test scores.

Test	Regression coefficient	95% CI	F statistic	Numerator *df*	Denominator *df*	*p*	Direction^a^
DRS-2	− 0.891	− 1.937 to 0.156	3.007	1	31.76	0.09	Virtual
MoCA	− 0.212	− 0.972 to 0.547	0.323	1	33.00	0.57	Virtual
Clock draw test	− 0.356	− 0.789 to 0.077	2.803	1	33.00	0.10	Virtual
Boston naming test	− 0.654	− 1.313 to 0.006	4.064	1	33.02	0.05	Virtual
Judgment of line orientation	− 0.905	− 2.689 to 0.879	1.066	1	33.00	0.31	Virtual
Trails A	− 3.942	− 9.503 to 1.618	2.092	1	30.83	0.16	In person
Trails B	6.674	− 9.796 to 23.144	0.739	1	15.85	0.40	Virtual
Symbol digit modalities test	− 1.511	− 6.025 to 3.002	0.464	1	33.00	0.50	Virtual
HVLT-R total immediate	0.629	− 1.225 to 2.483	0.478	1	32.00	0.49	In person
HVLT-R delayed	− 0.549	− 1.908 to 0.810	0.677	1	32.00	0.42	Virtual
HVLT-R recognition discrimination	0.193	− 0.617 to 1.003	0.236	1	32.00	0.63	In person
Letter-number sequencing	0.327	− 0.456 to 1.110	0.721	1	33.00	0.42	In person
Verbal fluency—FAS	− 2.229	− 5.180 to 0.722	2.361	1	33.00	0.13	Virtual
Verbal fluency—animals	− 1.776	− 3.138 to − 0.414	7.040	1	33.01	0.01	Virtual

^a^Direction indicates which administration type performed better.

### Overall retest reliability

We also assessed retest reliability for visit 1 versus visit 2 scores. ICCs found that, once again, only the DRS-2, Trails A, and phonemic verbal fluency tests demonstrated good retest reliability when administered 3–7 days apart, regardless of the mode of administration (Table 5). All other tests showed poor or moderate reliability.

**Table 5:** Intraclass correlations of test scores at visit 1 and visit 2.

Test	N	Visit 1 mean (SD)	Visit 2 mean (SD)	Correlation coefficient	Interpretation of reliability	95% CI
DRS-2	34	136.3 (5.5)	137.0 (5.7)	0.849	Good	0.720–0.837
MoCA	35	26.6 (2.7)	27.1 (2.3)	0.610	Moderate	0.357–0.781
Clock draw test	35	5.1 (1.2)	5.6 (0.8)	0.179	Poor	− 0.117 to 0.462
Boston naming test	35	57.2 (3.0)	58.4 (2.5)	0.691	Moderate	0.389–0.845
Judgment of line orientation	35	22.6 (5.5)	24.9 (3.6)	0.350	Poor	0.044–0.602
Trails A	32	44.3 (19.3)	44.5 (24.5)	0.752	Good	0.549–0.871
Trails B	19	77.2 (34.2)	78.8 (44.5)	0.684	Moderate	0.339–0.866
Symbol digit modalities	35	35.5 (14.9)	33.7 (12.7)	0.560	Moderate	0.284–0.750
HVLT-R total immediate	35	21.9 (4.6)	21.8 (5.6)	0.513	Moderate	0.217–0.722
HVLT-R delayed	35	7.3 (3.2)	6.5 (3.8)	0.424	Poor	0.118–0.658
HVLT-R recognition discrimination	35	9.5 (1.8)	9.0 (2.2)	0.410	Poor	0.102–0.649
Letter-number sequencing	35	9.1 (2.2)	9.5 (2.0)	0.417	Poor	0.106–0.655
Verbal fluency—FAS	35	46.3 (13.1)	47.3 (15.3)	0.814	Good	0.664–0.902
Verbal fluency—animals	35	19.6(5.5)	19.5 (6.3)	0.739	Moderate	0.540–0.859

Examining the impact of visit 1 versus visit 2 on cognitive test performance in linear mixed-effects models while accounting for administration type, Clock Draw (*p* = 0.02), BNT (*p* = 0.001), and JLO (*p* = 0.01) performance were significantly better at the second visit compared with visit one.

## Discussion

As telemedicine becomes more widely used, both clinically and in clinical research, the need to administer cognitive testing virtually has grown. We found that in PD overall cognitive test performance was similar when administered virtually versus in-person, but that there is significant variability in test performance over the short-term regardless of the mode of administration.

Average cognitive test scores for virtual testing were similar to in-person testing, and in the linear mixed-effects models, mode of administration did not predict test performance, except for better performance for several tests when administered virtually. However, the retest reliability for virtual versus in-person testing was poor to moderate for most tests, which prompted us to examine overall retest reliability (i.e., visit 1 versus visit 2). The findings were similar, with most tests showing poor retest reliability from visit 1 to visit 2, separated by just 3–7 days. A mix of oral and visual testing was conducted with no differences in reliability between those testing types, but using oral versions of certain tests (e.g., Trails B) can help overcome significant technical issues when trying to administer some tests virtually.

The results suggest that there are significant short-term fluctuations in cognitive performance in PD patients, which has implications for interpreting a single or one-time test score in the context of clinical care and clinical research. Cognitive fluctuations can occur in PD patients who are being treated with levodopa to manage their motor symptoms, as part of non-motor fluctuations^37^. In addition, fluctuations in cognition, attention, and arousal are a core clinical feature for the diagnosis of dementia with Lewy bodies^38^, a disorder related to PD. On a clinical questionnaire about PD features, 15 (42.9%) of our participants self-reported experiencing cognitive fluctuations that could explain, in part, the variability in test scores we found in such a short period of time. The reasons for low retest reliability could differ between those participants with and without a diagnosis of cognitive impairment, but our small sample size prevented such secondary analyses. Due to small sample sizes and difficulty recruiting, further analyses of cognitive subgroups could not be conducted with meaningful results. Future testing with larger cohorts and a wide range of cognitive abilities will help determine which PD patients are most appropriate for virtual cognitive testing, as well as the effect of cognition. Alternatively, some of the low retest reliability that we found may be inherent to the tests themselves.

None of the variables included in the linear mixed-effects models (i.e., administration type, visit number, sex, age at test, PD duration, and education) had a significant effect, except visit number on the BNT, JLO, and Clock Draw, which may have been due to the practice (i.e., learning) effects. Most participants participated in the core study for many years and are familiar with these tests, thus we would not expect a practice effect at this point. To our knowledge, parallel versions of the BNT and Clock Draw are not available. While odd–even short forms of the JLO exist, only the odd items of the JLO were administered in our study. Alternate versions of the MoCA were considered but were not utilized, in part as only version 7.1 was utilized in the parent study at the time of this sub-study initiation.

Virtual administration of testing introduced novel limitations not normally seen with in-person administration. Unavoidable technological issues, such as limited internet connectivity and audiovisual issues were problematic for some participants. Screen size was limited by the participants’ choice of device, reflecting the variability of devices used in the general population, and smaller screen sizes may have influenced performance on tests that largely rely on visual stimuli (e.g., JLO, BNT and MoCA). Larger sample sizes using a range of devices and screen sizes, and examining results by type of device, are needed to evaluate this effect. Aging populations tend to have advanced impairment compounded by additional comorbidities that can lead to trouble understanding and troubleshooting computers or similar technology, sometimes requiring patience and thorough instructions. Additionally, older populations tend to have more difficulty with hearing, which is only exacerbated by the slightly muffled audio quality on video chats despite increased volume settings. Finally, there were three instances of suspected “cheating” at virtual visits. Although unconfirmed, it was suspected that one patient may have completed the SDMT in their virtual testing packet before or after the formal testing session, despite being instructed not to open the test packet until the time of test, and the other two participants may have written down the words on the HVLT-R for recall during administration. The Trail Making Test proved incompatible with virtual administration for some participants, as raters were unable to directly observe participants completing the tests and were therefore unable to correct them as required by administrator test instructions. Attempts were made to angle participants’ cameras to observe their work directly, but this proved to be impractical for both participants and administrators. This occurred in 3 (8.6%) participants for Trials A and 16 (45.7%) participants for Trails B. For this reason, the oral versions of these tests may prove more useful in a virtual setting than the traditional written version.

Virtual administration of cognitive tests is limited to those who have reliable internet access and technology that can support video conferencing. Also, we did not attempt virtual testing with patients with moderate-severe dementia, as we did not think it would be feasible to assess the participants effectively. Additionally, a certain degree of computer literacy is required, which is especially a problem in older, cognitively impaired cohorts. However, our cohort was highly educated, and likely has a level of computer literacy and access to high-quality devices and internet connectivity, so our research may not be generalizable to the broader PD community. Also, while cognitive testing in a clinical setting tends to be personalized to the participant’s needs, this battery was fixed and part of a pre-existing, long-standing study. Despite this, a virtual option makes cognitive testing much more accessible for non-local participants, and especially so for PD patients with advanced motor disabilities. Raters were limited in their ability to accurately or immediately score some tests, such as the Clock Draw Test and Trail Making Test, until the test packet was returned via mail by participants, instead of relying on screenshots taken during testing. Thus, obtaining accurate data was dependent on both the patient and the mail to return the packets, though this did not prove to be an issue for our cohort. Scheduling constraints prevented the two visits from being conducted at the same time of day for each participant, although all participants were evaluated in an “on” state by self-report. Finally, we did not have a comparison group of demographically-comparable healthy controls to determine if our observed suboptimal reliability is unique to PD.

While traditional in-person paper and pencil testing with a trained neuropsychologist remains the gold standard, there may be situations in which virtual testing is necessary or helpful. This study provides preliminary evidence that virtual administration of cognitive testing may produce similar results to traditional in-person testing for numerous global and detailed cognitive tests at the group level. However, multiple issues that arose throughout the course of this study raise questions about the feasibility of virtual cognitive testing in PD. In a somewhat unexpected additional finding, there was significant short-term variability in cognitive test performance overall, regardless the mode of administration, which has implications for interpreting cognitive test results from a single session administered as part of clinical care or clinical research. Future studies with larger sample sizes, including patients with a wider range of cognitive abilities (a mix of normal cognition, MCI, and dementia), and conformity with device type and familiarity with device utilized are needed to further evaluate virtual testing as a possible substitute for traditional in-person testing, and to explain variability in performance. Regardless of any limitations, in a typically older population for which in-person clinical or clinical research visits can be a challenge, virtual cognitive testing in PD merits further study.

### Supplementary Information

Supplementary Information.

## Data Availability

The datasets used and/or analyzed during the current study available from the corresponding author on reasonable request.
