# Supplementary material for: Validating virtual administration of neuropsychological testing in Parkinson disease: a pilot study
Source: Sci Rep. 2023 Sep 27;13:16243. doi: 10.1038/s41598-023-42934-0 (PMC10533878; doi:10.1038/s41598-023-42934-0)
Supplement: Supplementary file 1 — Supplementary Information. [file 41598_2023_42934_MOESM1_ESM.pdf]

Validating virtual administration of neuropsychological testing in Parkinson disease: a pilot study

Julia Gallagher, BS[a], Eugenia Mamikonyan, MS[b], Sharon X. Xie, PhD[c], Baochan Tran, PsyD[a], Sarah Shaw[a], and Daniel Weintraub, MD[a,b,d,*]

Supplementary Material: Virtual Test Packet

DRS Graphomotor Design 1

DRS Graphomotor Design 2-4

DRS Construction Designs 1-2

DRS Construction Designs 3-6

FORM I KEY SUBJECT ID ______________

)	Γ	∸	(	+	⊣	>	⊢	⨪
1	2	3	4	5	6	7	8	9

(	⊣	⨪	(	⊢	>	⨪	Γ	(	>	⨪	(	>	(	⨪

Γ	>	(	⨪	⊣	>	⊢	Γ	(	⨪	>	∸	Γ	⊢	)

Γ	⊣	+	)	(	⊢	+	Γ	)	⊣	⨪	∸	⊢	Γ	+

⨪	Γ	⊣	(	>	Γ	(	⊣	>	+	∸	)	⊢	>	Γ

∸	⊣	)	⊢	>	+	Γ	⊣	⨪	⊢	+	∸	⨪	)	(

>	∸	+	⨪	⊢	>	Γ	∸	(	+	⨪	⊣	>	)	Γ

⨪	)	+	∸	⊢	+	)	⊣	(	∸	⨪	(	Γ	⊢	>

⊣	⨪	(	>	Γ	⨪	(	>	∸	+	⊢	⊣	Γ	)	∸

TOTAL CORRECT ☐

Material from the copyright © 1973, 1976, 1982 by Western Psychological Services. Format adapted by N. Hunter, University of Rochester Medical Center, for specific, limited research use under license of the publisher, WPS, 12031 Wilshire Boulevard, Los Angeles, California 90025, U.S.A. (www.wpspublish.com). No additional reproduction, in whole or in part, by any medium or for any purpose, may be made without the prior, written authorization of WPS. All rights reserved.

CLOCK DRAWING TEST

(command)

SKIP

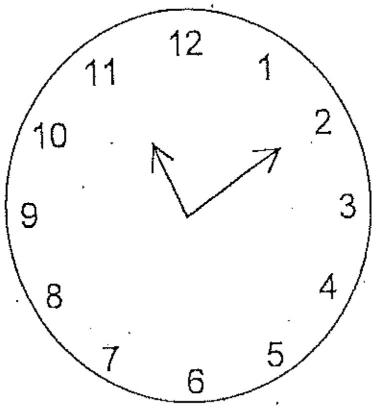
12
1
2
3
4
5
6
7
8
9
10
11

TRAIL MAKING

Part A

SAMPLE

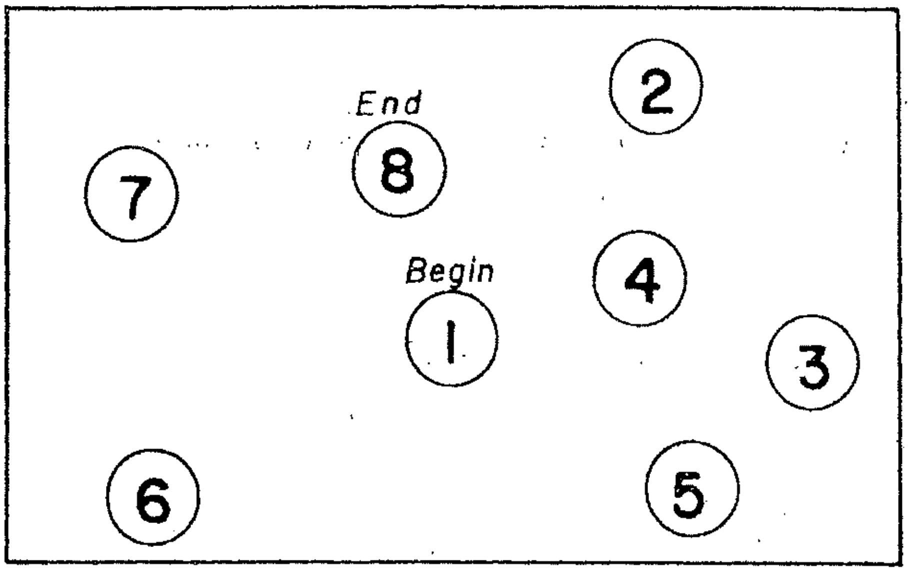

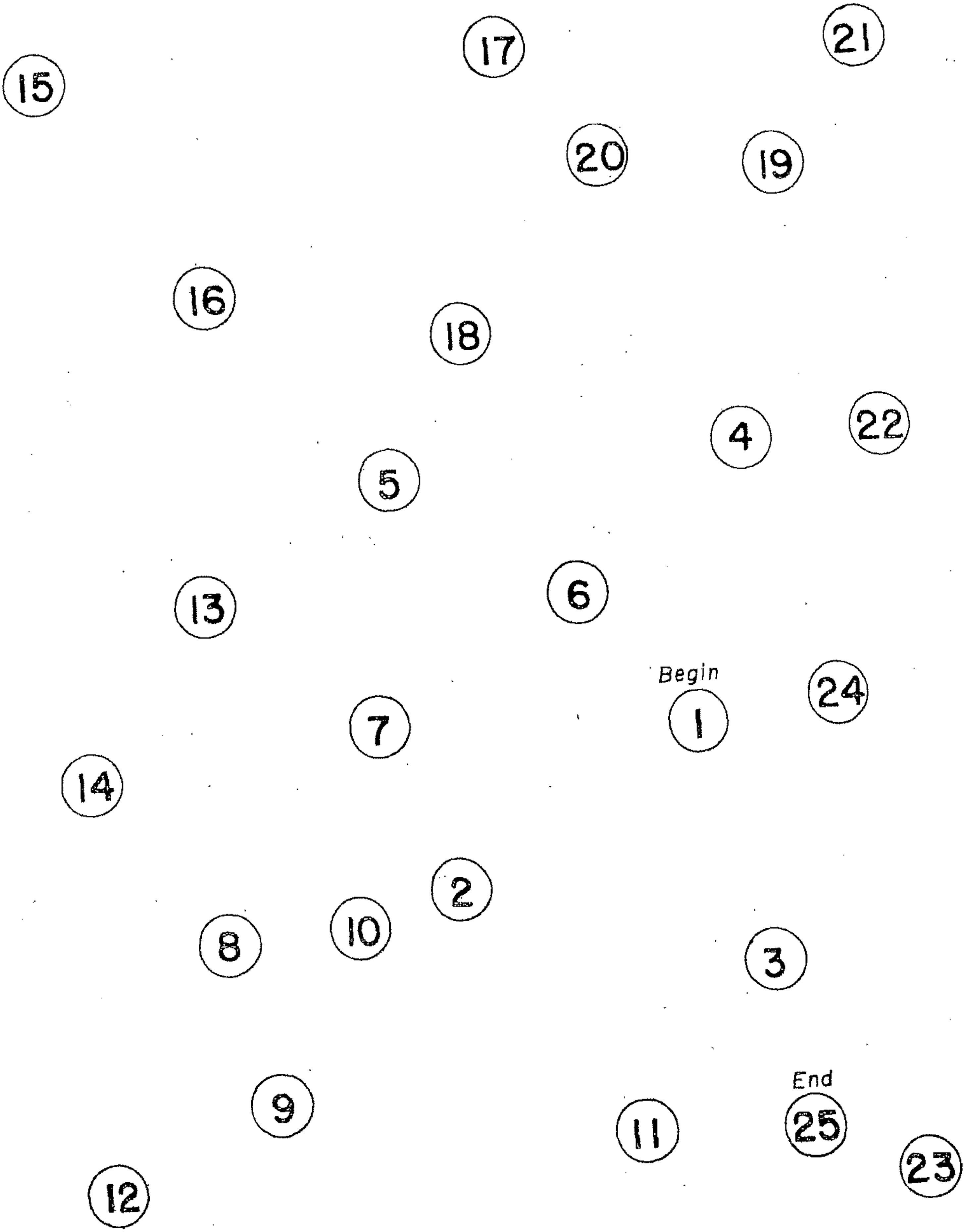

15
17
21
20
19
16
18
4
22
5
6
13
Begin
1
24
7
14
2
10
8
3
End
9
11
25
23
12

TRAIL MAKING

Part B

SAMPLE

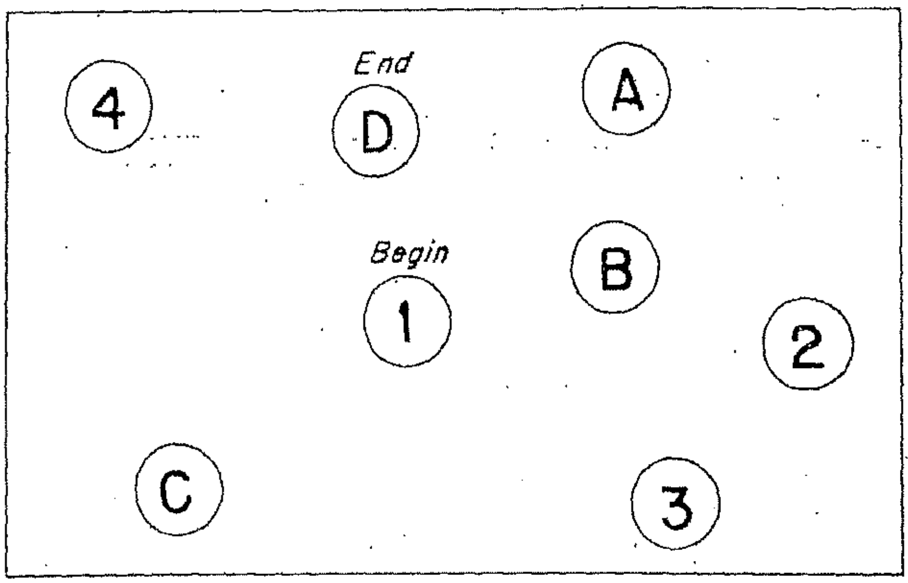

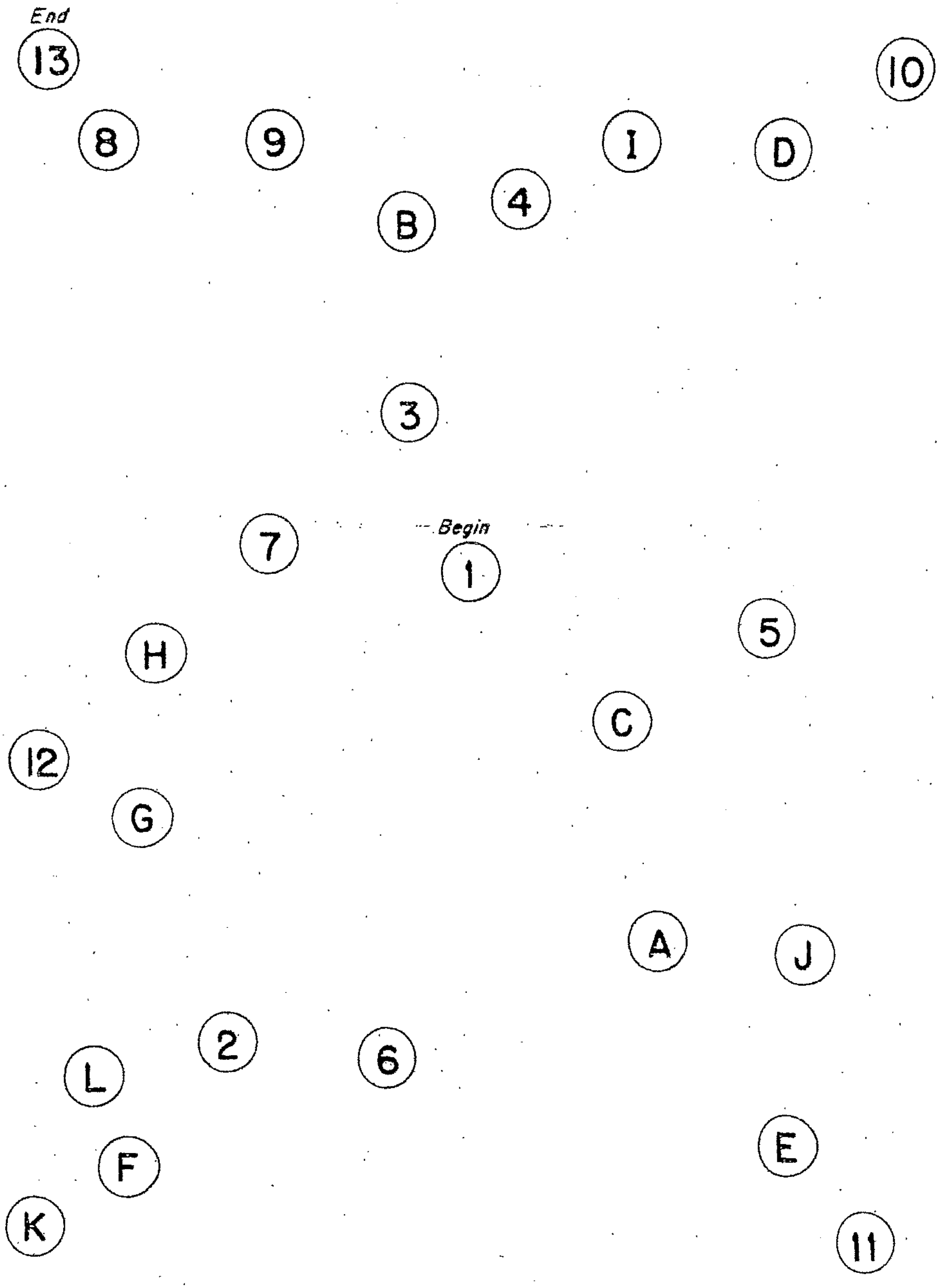
End
13
10
8
9
I
D
4
B
3
Begin
7
1
5
H
C
12
G
A
J
2
6
L
E
F
K
11

Montreal Cognitive Assesment (MoCA)

VISUOSPATIAL/EXECUTIVE

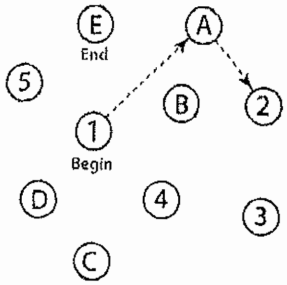

[ ]

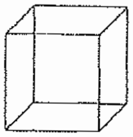

Copy cube

[ ]
